# The Neuropsychology of Starvation: Set-Shifting and Central Coherence in a Fasted Nonclinical Sample

**DOI:** 10.1371/journal.pone.0110743

**Published:** 2014-10-22

**Authors:** Sarah Pender, Sam J. Gilbert, Lucy Serpell

**Affiliations:** 1 Research Department of Clinical, Educational, and Health Psychology, University College London, London, United Kingdom; 2 Institute of Cognitive Neuroscience, University College London, London, United Kingdom; 3 Eating Disorders Service, North East London Foundation Trust, Essex, United Kingdom; Hospital Universitario de La Princesa, Spain

## Abstract

**Objectives:**

Recent research suggests certain neuropsychological deficits occur in anorexia nervosa (AN). The role of starvation in these deficits remains unclear. Studies of individuals without AN can elucidate our understanding of the effect of short-term starvation on neuropsychological performance.

**Methods:**

Using a within-subjects repeated measures design, 60 healthy female participants were tested once after fasting for 18 hours, and once when satiated. Measures included two tasks to measure central coherence and a set-shifting task.

**Results:**

Fasting exacerbated set-shifting difficulties on a rule-change task. Fasting was associated with stronger local and impaired global processing, indicating weaker central coherence.

**Conclusions:**

Models of AN that propose a central role for set-shifting difficulties or weak central coherence should also consider the impact of short-term fasting on these processes.

## Introduction

### Set-Shifting

Set-shifting is the ability to move flexibly between different tasks or mental ‘sets’. Set-shifting difficulties may manifest as cognitive or behavioural inflexibility, for example, rigid approaches to problem solving or difficulties managing dynamic social interactions. Set-shifting deficits have been shown in a number of psychiatric disorders including unipolar depression [1], [2], bipolar disorder [3], schizophrenia [4], [5] and eating disorders [6]. Studies of Anorexia Nervosa (AN), Bulimia Nervosa (BN) and obese individuals have revealed set shifting abnormalities on a range of different tasks [6]–[9]. Some set-shifting impairments have also been reported in first degree relatives of those with AN. Thus, set-shifting difficulties have been proposed as a possible endophenotype for AN [10].

### Central Coherence

Another potential endophenotype for AN is weak central coherence [11]. The concept of weak central coherence was developed as a description of a cognitive style associated with autism spectrum disorders (ASD; see [12]). Clinically, weak central coherence is associated with paying attention to details, alongside difficulties integrating global concepts into a broader understanding. For example, an individual with AN who focuses intently on the details of exactly what she has eaten and finds it difficult to consider the long-term health consequences of starvation may be viewed as having weak central coherence.

A systematic review of central coherence in eating disorders (EDs) reported that individuals with AN and BN showed poorer performance on tasks requiring global or ‘bigger picture’ processing than HVs [11], [13]. Studies also suggest that those with AN are better at tasks requiring detail focus [14], [15].

### The Role of Starvation

Individuals with AN are severely undernourished, characterised by a combination of short-term food restriction and chronic undernourishment [16]. Those with AN restrict their food intake significantly, skipping meals, eating smaller portions, and lower calorie foods [16]. Individuals with AN experience specific structural and metabolic brain changes [17], [18] compared with HVs, probably due to prolonged malnutrition. In healthy individuals, prolonged food restriction is associated with difficulties such as stereotypic and obsessive rituals [19] which appear largely reversible. In some cases, unintentional weight loss can trigger AN [20], [21], which suggests a facilitative role of starvation. A recent review of the impact of fasting on cognition [22] provided evidence that even relatively short periods of fasting can disrupt a range of cognitive processes including set-shifting. Thus, any model proposing a role of cognitive rigidity and/or weak central coherence in the development and maintenance of AN should also consider how far this may be a consequence of, rather than a cause, of, starvation [23].

Studies investigating the impact of starvation on set shifting difficulties in eating disorders have had mixed findings. Most studies show no correlations between set-shifting difficulties and Body Mass Index (BMI) [24]–[26], although the relationship between BMI and acute starvation is not clear. Some studies have shown that set shifting deficits improve with recovery from AN [27], [28] and are related to the degree of fasting [26], [29]. However, other studies have shown that set shifting deficits persist following recovery from AN [30], [31]. Studies of central coherence have indicated that individuals recovered from AN have an intermediate profile between acute AN and HVs [14], [32], suggesting food deprivation may play a role.

### Current Study

Set shifting difficulties and weak central coherence in EDs present a promising avenue for ED research, however, it is not yet clear which factors might influence deficits. Research designs are often confounded by patients' current AN status, making it impossible to separate the situational effects of food restriction from the more enduring features of the ED.

In this study, a repeated measures design was used to examine the effect of short-term fasting on tasks measuring set-shifting and central coherence in HVs. Although this is not the same as the chronic starvation experienced by those with AN, it may provide insight into the relationship between one aspect of eating behaviour observed in AN, i.e., short-term food deprivation, and cognitive functioning in those with AN. If short-term fasting in HVs leads to a cognitive profile that is comparable to that observed in AN, this would provide a case for further investigation of the role of short- and long-term starvation in the development and maintenance of AN.

This study aimed to replicate findings from a previous study [33], which found that in HVs, short-term fasting exacerbates set-shifting difficulties on a rule-change task. Based on these findings, it was expected that fasting should increase the difference in reaction time (RT) for shift trials vs. stay trials.

The effect of fasting on central coherence was also explored. In line with research showing that individuals with current AN show greater deficits in central coherence than those recovered from AN [14], [32] this study hypothesised that short-term fasting would intensify any local processing bias, leading to improved detection of local stimuli and impaired detection of global stimuli on a computerised task [34] and faster detection of hidden figures on a paper and pencil task [35].

Depression moderates impaired set-shifting in AN [30]. It was expected that low mood would be associated with greater set-shifting difficulties. Finally, higher levels of self-reported perseveration and ED pathology were expected to be linked to poorer performance on set-shifting and central coherence tasks.

## Materials and Methods

### Ethics

This study was approved by the local research ethics committee and all participants provided written informed consent before taking part. Participants were given an information sheet detailing the possible risks of fasting and advised to stop fasting immediately if feeling unwell.

### Participants

For a moderate effect size at an alpha level of 5% with a power level of 80%, a sample size of 34 was required. Participants were 60 women who volunteered in response to a poster on the university campus or a web-based advertisement. Individuals were suitable if they reported themselves to be fit and healthy, female, aged between 18 and 30 years, and fluent English speakers. Participants were excluded if they reported a lifetime history of any psychiatric illness, or if they knowingly had any medical condition that would make fasting dangerous, such as diabetes or pregnancy. Participants completed self-report measures of current anxiety, depression and eating pathology. All scores on these measures were within one standard deviation of published group norms for community samples [36], [37] indicating that the current sample is at least comparable on measures of anxiety, depression and eating pathology to a broader community sample. Participants were paid a nominal amount or received university course credits for their participation.

The mean age of participants was 23.2 years (SD = 3.8, range 18–29) and the mean BMI was 22.3 kg/m2 (SD = 3.5, range 16.4–32.2). Two participants had a BMI <17.5 kg/m2, below the clinical cut-off for AN, but were included as their scores on a measure of eating pathology were within one standard deviation of the group mean score, indicating that although they had low body weight, their self-reported eating behaviours were similar to the reported eating behaviour in the group as a whole. Furthermore, eating pathology scores in the current study were within one standard deviation of scores from two healthy female community samples [37], [38], indicating that there was a similar level of ED behaviour as reported in other HVs. All participants in the sample were educated to university level. Participants were instructed to fast for 18 hours prior to one of the testing sessions; on questioning by the researcher at the fasting session, all participants reported that they had done so. During the fasting period, participants were encouraged to consume water but required to abstain from alcohol and sugary drinks.

### Measures

#### Height/weight

Height and weight were measured using a portable stadiometer and a digital weighing scale accurate to within 0.05 kg.

#### Mood and anxiety

Participants rated their mood and anxiety at each session using the Hospital Anxiety and Depression Scale (HADS; [39]). This 14-item self-report measure yields subscale scores for anxiety and depression, with higher scores indicating more symptoms and higher distress. The HADS is widely used in research and clinical settings and has acceptable validity in community samples [39].

#### ED symptoms

The Eating Disorder Examination- Questionnaire-6 (EDE-Q6; [38]) is a 28-item self-report questionnaire. The EDE-Q6 yields four subscale scores (dietary restraint, shape concern, weight concern, and eating concern) and a global score. A high level of agreement has been reported in assessing the core features of ED pathology in the general population between the gold standard diagnostic interview (the EDE), and the EDE-Q [37], [40].

#### Perfectionism, persistence, and perseveration

The 22-item self-report Perfectionism, Persistence and Perseveration Questionnaire (PPPQ-22; [41], [42]) asks participants to consider the past few weeks as they rate each statement (e.g., ‘When shopping in the supermarket, I walk down the aisles one by one until I have covered the whole store, even if I only need a couple of items’) on a 5-point scale according to how much they endorse it (1 =  not at all true of me and 5 =  totally true of me). The PPPQ-22 has adequate test-retest reliability (r = .73 for perfectionism, r = .79 for perseveration and r = .89 for persistence, all p<.001) and internal consistency (Cronbach's α = .70 for perfectionism,.64 for perseveration, and.76 for persistence) in a nonclinical sample [41].

### Experimental Tasks

#### Rule-change task

**Figure 1:**
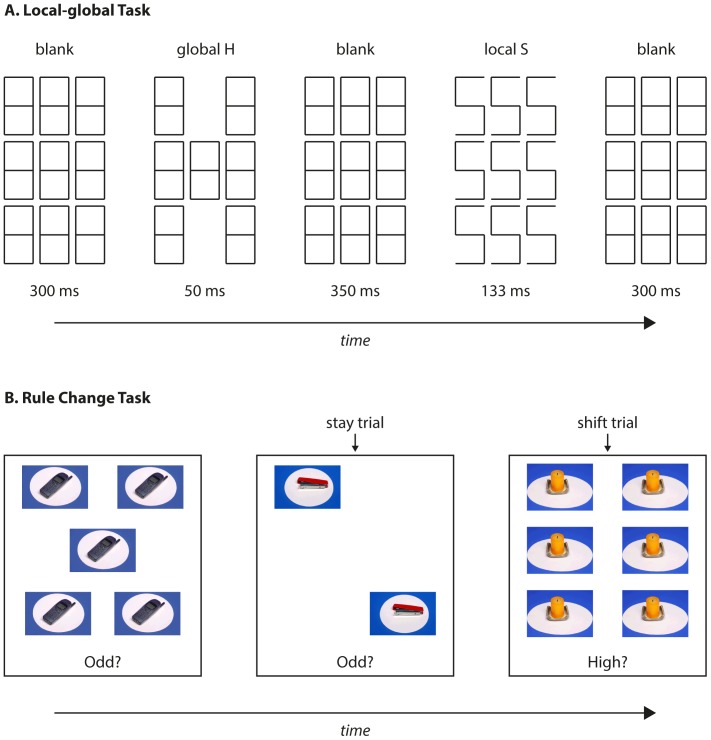
Schematic illustration of the rule change and local-global tasks.

This task was based on the task used by Bolton et al. [33]. However, the current study used only non-food images, given that no differences were found between food and non-food items in the previous study. On each trial, participants were asked to judge the number of photographs presented on a computer screen (Figure 1). Between one and six identical photographs of everyday items were presented. Participants were asked to respond with one of two computer keys to indicate Yes or No to one of four questions: ‘Odd?’ (i.e. 1, 3, or 5 items); ‘Even?’ (i.e. 2, 4, or 6 items); ‘High?’ (i.e. 4, 5, or 6 items); or ‘Low?’ (i.e. 1, 2, or 3 items). Participants were asked to respond as quickly and as accurately as possible. On two thirds of trials the question presented was the same as the previous trial; these were ‘stay trials’. On the remaining trials one of the other questions was randomly selected; these were ‘switch trials’. On each trial, the stimuli remained on screen until a response key was pressed, followed by a random delay of 250–500 ms before the next set of stimuli. Participants were not given the opportunity to practise the task prior to testing, and were administered one block of 100 trials (note: in the Bolton et al., 2014 study there were 20 practice trials and 400 experimental trials; the present procedure was otherwise identical to the earlier study). Shift costs were defined as the mean difference in RT between “shift” and “stay” trials: a higher shift cost indicated greater difficulty in changing response from one type of question to another.

#### Local-global processing task

This measure was based on a task used to measure central coherence in participants with ASD [34] It has recently been used in a clinical population [35]. Potential stimuli consisted of the five letters E, H, P, S, and U, which could be presented in either a ‘local’ or a ‘global’ configuration (Figure 1). Each trial consisted of two consecutive letters, separated by a blank display for 350 ms, and with blank displays presented for 300 ms before and after each trial. At the end of each trial, participants were asked to make an unspeeded response to identify the two letters by pressing the corresponding keys on the computer keyboard.

Participants completed four blocks of trials in the following order; two global letters (GG), two local letters (LL), a global then a local letter (GL), a local then a global letter (LG). Each block consisted of 5 slow practice trials (with letters presented for 300 ms) followed by 25 trials in which global letters were presented for 50 ms and local letters for 133 ms. After each trial participants received feedback on the correct answer. The proportion of correct trials for both the first and the second letter in each block of trials was calculated.

#### Group embedded figures task (GEFT)

The GEFT is a pencil-and-paper task commonly used as a measure of central coherence [43]. The GEFT measures the time taken to find one of eight simple shapes in 18 complex figures. In this study, reliance on memory was eliminated as a picture of the simple shapes was displayed beside the complex design. A similar methodology has been used to examine central coherence in participants with AN (e.g. [14]). Faster completion times and fewer false claims are associated with stronger local processing [14].

### Procedure

The study used a within-subjects repeated measures design to compare individuals' scores on tests of set-shifting and central coherence at two time points; when participants had fasted for 18 hours, and when they were satiated. Order of fasting and satiated trials was counterbalanced between participants. Mean time between the two sessions was 10.82 days (SD = 3.98, range 5–21).

Participants were encouraged to be open with the researcher about whether they had fasted as instructed, and all participants reported that they had done so. In order to encourage adherence to fasting, participants were informed that a subsample would be randomly selected to give a urine sample to check ketone levels. However, as ketone levels are not a reliable measure of fasting after just 18 hours [33], no samples were taken. Each participant attended two sessions of one hour, which differed only slightly from one another. At both, participants were asked to complete the HADS, followed by the GEFT, the rule-change task and then the local-global processing task. Task order was identical at each session. At the satiated session, participants also completed the EDE-Q6 and their height and weight was measured. After the second session, participants were paid and debriefed.

## Results

### Self-Report Measures

Mean anxiety (6.40, SD = 2.93, range 0–15) and depression scores (2.83, SD = 2.21, range 0–11) were within one standard deviation of HC norms [39]. All EDE-Q scores were within one SD of HC norms [37], [40]. BMI was not significantly correlated with EDE-Q subscales.

### Experimental Tasks

For each task, participants with scores greater than three standard deviations from the group mean in any cell of the experimental design were excluded as these scores indicate extreme outliers. Thus, the number of participants included in each analysis differed. The order of fasting and satiated sessions was entered into each ANOVA as a between-subjects factor in order to remove variance related to the effect of practice on test performance. Seeing as the order of fasting sessions was perfectly counterbalanced between participants, effects of the fasting/satiated manipulation were uncounfounded from practice effects.

#### Rule-change task

Only correct trials with RTs between 150 and 3000 ms were included in the analysis in order to eliminate trials on which participants were inattentive or responded prematurely. Data for 58 participants were analysed.

The analysis examined shift cost rather than question content, therefore data were averaged over the four tasks to yield four scores for each individual: fasting shift RT (M = 1451 ms, SD = 281, range 701–2355), fasting stay RT (M = 1182 ms, SD = 206, range 648–1788), satiated shift RT (M = 1488 ms, SD = 299, range 888–1974), satiated stay RT (M = 1258, SD = 270, range 710–1867).

A 2×2 (condition [fasting, satiated] x trial-type [stay, shift]) repeated measures ANOVA showed that participants responded more quickly when fasting than satiated, F(1, 56) = 6.23, p = .016. There was also a main effect of trial-type, F(1, 56) = 284.8, p<.001; participants responded more slowly on shift than stay trials. There was a marginally-significant Fasting x Trial-type interaction, F(1, 56) = 3.96, p = .052, indicating that there was a larger difference in RT between stay and shift trials in the fasting than in the satiated condition, despite the overall faster RT. When the cost of switching was calculated as a proportion (i.e., RTshift/RTstay) rather than an absolute difference (i.e., RTshift – RTstay), this cost was significantly greater in the fasting than the satiated condition, F(1, 56) = 5.3, p = .025.

#### Local-global processing task

The proportion of correct responses for each trial type was used as a measure of performance. Only trials with a correct response to the first letter were included in the analysis of the second letter (as suggested in [34]). Data for 58 participants were analysed.

**Figure 2:**
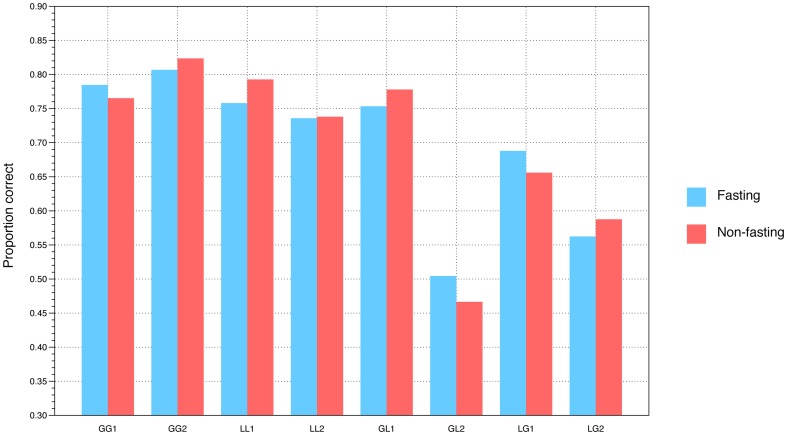
Proportion correct in each condition of the local-global task, separately for the fasting and non-fasting conditions. Results are separated into the first and second letter in each condition, e.g. GL1 would be the initial global letter and GL2 the subsequent local letter in a GL trial. Note that the GL1, GL2, LG1, and LG2 bars all show that performance is superior for local letters in the fasting condition and global letters in the non-fasting condition.

A 2×4×2 (Condition [fasting, satiated] x Trial-type [global-global, local-local, global-local, local-global] x Letter position [first, second]) ANOVA examined the effect of fasting, trial-type, and letter position on performance. Fasting did not affect overall proportion of correct responses, F(1, 56)<0.1, p = .94. There was a highly significant effect of Trial-type, F(3, 54) = 108, p<.001. Performance was markedly more accurate in global-global and local-local conditions than in the global-local or local-global conditions (see Figure 2). There was a significant Condition x Trial-type x Letter position interaction, F(3, 54) = 3.9, p = .014, indicating that fasting affected performance in a way that differed across trial-type and letter position. In the global-local and local-global conditions, which required a switch between global and local configurations, accuracy was higher for local letters in the fasting condition and global letters in the satiated condition.

This interaction was explored using two separate 2×2 (Condition [fasting, satiated] x Trial-type [global-local, local-global]) ANOVAs to examine responses to Letters 1 and 2 in the two conditions. For Letter 1 there was a significant Fasting x Trial-type interaction F(1, 56) = 6.7, p = .012; this interaction approached significance for Letter 2 F(1, 56) = 3.3, p = .076. Taken together, these results suggest that fasting is associated with a bias towards local processing and impaired global processing when the task involves switching between local and global perspectives.

#### Group embedded figures task

Median time taken and number of false claims were taken as measures of task performance. Data for 56 participants were analysed. The average time taken to complete a figure was 11.97 s (SD = 5.95, range 3–28 s) when fasting, and 13.7 s (SD = 6.30, range 3–27 s) when satiated. The mean number of false claims was 1.86 (SD = 1.38, range 0–5) when fasting and 2.63 (SD = 2.01, range 0–8) when satiated.

Two separate general linear models were used to analyse the effects of fasting on completion times and number of false claims. There was a trend towards faster completion times in the fasting condition, F(1,54) = 2.98, p = .09. Participants made significantly fewer false claims when fasting than when satiated, F(1,54) = 5.42, p = .024.

#### Additional analyses

Correlational analyses examined relationships between mood, self-report perseveration, and eating pathology and experimental task performance. No significant associations were noted.

## Discussion

### Main Findings

This study aimed to explore the effect of short-term fasting on tasks measuring set-shifting and central coherence. Consistent with the finding of Bolton and colleagues [33], on the rule-change task there was a greater cost of switching rules in the fasting compared with the satiated condition. On a local-global processing task, short-term fasting was also associated with superior local and impaired global processing, indicative of weaker central coherence when fasting. Consistent with this finding, there was a trend towards faster detection of embedded figures in the Group Embedded Figures Test when participants were fasting, along with a reduced error rate. The hypotheses that lower mood, higher ED symptomatology, and higher self-reported perseveration would be associated with set-shifting difficulties were not supported. Seeing as the selective effects of fasting were associated with improved performance in some conditions, the present results are unlikely to relate to preoccupation by hunger or distraction in the fasting condition.

### Central Coherence

ED research suggests that AN is associated with weak central coherence [11], [13], [14] however, as food deprivation is inevitably correlated with AN, it is hard to disentangle whether such a bias is a core feature of AN, a side-effect of short-term starvation, related to more chronic undernourishment, or a combination of the three. This study suggests that short-term fasting in HVs is associated with stronger local and weaker global processing on tasks measuring central coherence. It is possible that even if individuals with EDs do not display these kinds of patterns in information processing premorbidly, short-term food deprivation could be associated with this information processing style. Such an information processing bias might lead to cognitive inflexibility, for example, a tendency to focus on details and a difficulty ‘zooming out’ to consider the bigger picture. In turn, this processing bias could maintain some of the core clinical features of AN, for example, a focus on calorific information and a difficulty incorporating information about the long-term health consequences of starvation.

### Set-Shifting

A variant of the rule-change task used in this study has been used previously with a similar population [33]. The current study did not replicate the previous finding that overall performance was slowed by fasting; instead, mean response times were faster in the fasting condition. This may relate to differences from the earlier study in the presentation of fewer trials, involving non-food items only. It did however replicate the finding that fasting increased the shift cost, suggesting that while fasting did not impair overall task performance, it led to a selective difficulty in the ability to shift set. Thus, findings from both the current study and the earlier study of Bolton et al. [33] suggest that at least some of the difficulties in set-shifting reported in those with current AN could be accounted for by the effects of short-term fasting.

### Limitations

The study relied on participant honesty to abstain from food for 18 hours. A limitation is that as urinalysis is an insufficiently sensitive measure of short-term fasting, there was no way to verify whether participants had fasted as instructed. Future studies should investigate other ways of verifying fasting, for example, blood glucose. It is possible that some participants did not adhere to the fasting guidelines. However, significant differences between the fasting and satiated sessions indicate that enough participants abstained from food as instructed to demonstrate an effect. If some did not fast as required then the effects of fasting on performance may actually be underestimated.

The local-global processing task was designed to measure central coherence in individuals with ASD and has not previously been used in ED research, making the results from the current study difficult to compare with similar research investigating central coherence in EDs. However, findings from the current study do indicate some effect of short-term fasting on individual performance, suggesting that it is sensitive to cognitive local-global processing difficulties in HVs. Future ED research comparing performance on the local-global processing task with performance on other established measures of central coherence could elucidate whether this measure of central coherence is comparable to other measures.

In AN research it can be difficult to disentangle the effect of enduring traits from the effects of temporary states, such as between chronic undernourishment and short-term caloric restriction. Short-term fasting cannot fully replicate the effects of chronic undernourishment and hunger in AN. Chronic starvation leads to neurochemical, metabolic, and structural brain changes in patients with AN [16], [17]; alterations which often remain after recovery [27]. Fasting for eighteen hours does not lead to such changes. Furthermore, patients with AN often severely restrict food rather than completely abstaining from food. Findings from the paradigm used in the current study, with HVs who have fasted for a short period of time are certainly not directly comparable to research with participants with AN. This type of research may, however, begin to help researchers and clinicians to understand the role of starvation on cognitive performance. Future research comparing fasted HVs with AN patients is needed to further understand how changes in performance identified in this study compare to AN.

This was an exploratory study therefore multiple comparisons were necessary in order to explore possible interrelations between tasks and self-report data. Multiple correlations increase the likelihood of a Type I error. Future research should replicate the current findings, using larger sample sizes to allow the use of more rigorous statistical analyses.

Finally, the current sample ranged in age from 18 to 30, in order to best represent the population affected by AN. No information on ethnicity was gathered in this study, and all participants were studying at university level. These factors limit the generalisability of these findings to well-educated, female populations, and it is unclear whether findings from the current study would be affected by educational attainment.

### Clinical and Theoretical Implications

This study suggests that short-term fasting interacts with local and global information processing, either by exacerbating an inherent tendency towards weak central coherence, or by eliciting weak central coherence in those that under normal circumstances do not display impaired global and enhanced local processing. It is possible that some people have weak central coherence and a tendency towards perseveration and thus are at risk for AN. For these individuals, once AN takes hold, short-term food deprivation could exacerbate such tendencies. For others who do not have these difficulties premorbidly, even short-term fasting, for example rigid dieting or physical illness, might trigger weak central coherence, and thus play a role in maintaining behaviours characteristic of AN. This may explain why AN can be precipitated by unintentional weight loss [20]. If supported by further research, the current findings may also suggest that initial weight restoration alone could ameliorate some of the cognitive symptoms of AN, for example, cognitive rigidity, and allow the flexibility needed to consider recovery, as well as the global processing needed to focus on higher-order goals and the future. They may also be relevant in understanding why very underweight patients with AN often find it difficult to fully engage in psychological therapy.

These tentative findings indicate that refeeding alone could improve some of the core cognitive symptoms of AN, in addition to specialist treatments targeting flexibility, such as Cognitive Remediation Therapy [44], [45].

### Conclusion

The present findings support the hypothesis that in HVs, short-term fasting is associated with impaired set-shifting and weak central coherence. They suggest that any models of AN proposing a central role for set-shifting difficulties or weak central coherence should also consider the impact of short-term fasting on these processes.
